# iNovo479: Metabolic Modeling Provides a Roadmap to Optimize Bioproduct Yield from Deconstructed Lignin Aromatics by *Novosphingobium aromaticivorans*

**DOI:** 10.3390/metabo12040366

**Published:** 2022-04-18

**Authors:** Alexandra M. Linz, Yanjun Ma, Samuel Scholz, Daniel R. Noguera, Timothy J. Donohue

**Affiliations:** 1 DOE Great Lakes Bioenergy Research Center, University of Wisconsin-Madison, Madison, WI 53726, USA; amlinz@wisc.edu (A.M.L.); mayj03@gmail.com (Y.M.); sfscholz@wisc.edu (S.S.); dnoguera@wisc.edu (D.R.N.); 2 Wisconsin Energy Institute, University of Wisconsin-Madison, Madison, WI 53726, USA; 3 Department of Civil and Environmental Engineering, University of Wisconsin-Madison, Madison, WI 53706, USA; 4 Department of Bacteriology, University of Wisconsin-Madison, Madison, WI 53706, USA

**Keywords:** metabolic modeling, aromatic metabolism, thermodynamics, lignin conversion

## Abstract

Lignin is an abundant renewable source of aromatics and precursors for the production of other organic chemicals. However, lignin is a heterogeneous polymer, so the mixture of aromatics released during its depolymerization can make its conversion to chemicals challenging. Microbes are a potential solution to this challenge, as some can catabolize multiple aromatic substrates into one product. *Novosphingobium aromaticivorans* has this ability, and its use as a bacterial chassis for lignin valorization could be improved by the ability to predict product yields based on thermodynamic and metabolic inputs. In this work, we built a genome-scale metabolic model of *N. aromaticivorans*, iNovo479, to guide the engineering of strains for aromatic conversion into products. iNovo479 predicted product yields from single or multiple aromatics, and the impact of combinations of aromatic and non-aromatic substrates on product yields. We show that enzyme reactions from other organisms can be added to iNovo479 to predict the feasibility and profitability of producing additional products by engineered strains. Thus, we conclude that iNovo479 can help guide the design of bacteria to convert lignin aromatics into valuable chemicals.

## 1. Introduction

Microbial production of chemicals from abundant renewable resources presents a potential cost-effective and sustainable alternative to current chemical production methods. One example of this alternative would be converting lignin, an under-utilized and abundant natural resource, to valuable products. Lignin is a polymer composed of aromatic subunits and accounts for a third of terrestrial carbon [1]. However, current methods for converting lignin-derived aromatics to valuable products are limited [2]. One major challenge in lignin valorization is its heterogeneity. The variety of aromatic subunit types and inter-subunit linkages in the polymer results in depolymerization mixtures containing many different aromatic compounds [3], creating a significant barrier to generating a single product from lignin. While these heterogeneous mixtures of substrates are challenging for valorization by chemical approaches, they can be less problematic for microbial conversion methods. The ability of some microbes to simultaneously catabolize multiple substrates makes them well suited for biological upgrading of depolymerized lignin because they can funnel the mixed aromatics to a single product [4,5].

We are studying the α-proteobacterium *Novosphingobium aromaticivorans* as a microbial chassis for producing valuable chemicals from lignin aromatics. Some traits that make *N. aromaticivorans* advantageous as a lignin-valorizing microbial chassis include co-metabolism of multiple aromatic and non-aromatic compounds, catabolism of aromatics from all three major monomeric units in lignin (guaiacyl (G), syringyl (S), and hydroxyl (H) units), metabolism of aromatic monomers and oligomers, and converging catabolic pathways that funnel mixtures of aromatic compounds into one or a small number of products, such as 2-pyrone-4-6-dicarboxylic acid (PDC) [6,7,8]. In addition, *N. aromaticivorans* is genetically tractable and can be handled using Biosafety Level-1 conditions. The ability of *N. aromaticivorans* to serve as a chassis for lignin valorization would be increased by predicting pathways and conditions needed to generate sufficient yields of valuable products.

In this study, we built iNovo479, a genome-scale metabolic model (GEM) for *N. aromaticivorans* that contains the major pathways for carbon metabolism, including characterized pathways for catabolism of aromatic compounds. We used iNovo479 to combine genome annotation and published experimental data on engineered strains to make predictions on aromatic metabolism, thermodynamic aspects of using different enzymes for aromatic compound catabolism, and the potential products that could be made from deconstructed lignin by engineered strains of *N. aromaticivorans*. The lessons learned from assembling iNovo479 as well as the predictions made by this GEM should be of interest to those interested in lignin valorization, aromatic metabolism, and optimizing metabolic processes in other bacterial chassis.

## 2. Results

### 2.1. Assembling iNovo479 as a GEM for N. aromaticivorans

iNovo479 was built using predicted KEGG annotations for the *N. aromaticivorans* DSM 12444 genome (hereafter referred to as *N. aromaticivorans*). The length of the *N. aromaticivorans* genome is 4.2 megabases. Genes were linked to metabolic reactions based on annotation data available in the Integrated Microbial Genomes database in 2019 [9]. Aromatic metabolism reactions were added to iNovo479 manually based on available knowledge in the literature, using custom IDs. Gap filling of reactions missing in key metabolic pathways was also performed manually. A summary of the contents of iNovo479 is provided in Table 1.

The biomass reaction of iNovo479 is a modified version of the aerobic biomass reaction in iRsp1095, the GEM assembled for another α-proteobacterium, *Rhodobacter sphaeroides* [10] (Dataset S1). Modifications were based on known differences between *N. aromaticivorans* and *R. sphaeroides,* as well as laboratory-determined biomass composition data on *N. aromaticivorans* (Table S1). A non-growth-associated maintenance reaction was also included in iNovo479.

### 2.2. Testing Predictions of Biomass Yields by iNovo479

**Figure 1:**
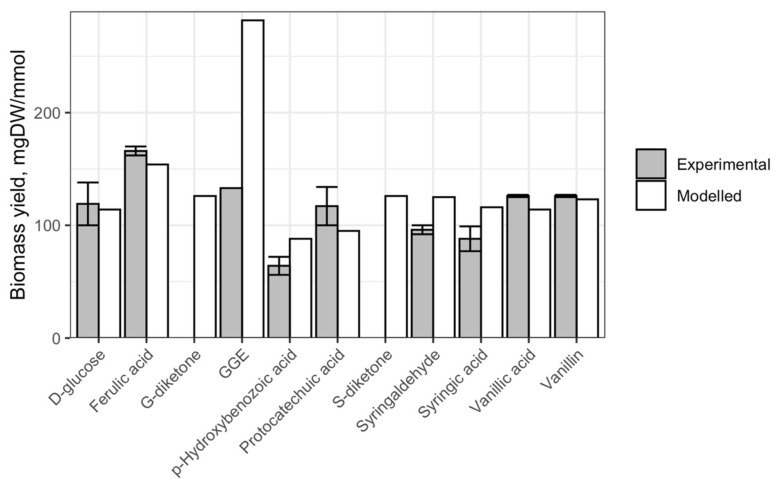
Predicted and experimentally determined biomass yields for *N. aromaticivorans* cultures grown on glucose or aromatic compounds. GGE = all isomers of guaiacylglycerol-β-guaiacyl ether; observed yield for GGE obtained from Kontur et al. [11]. G-diketone and S-diketone were not available to perform experimental yield measurements.

We sought to evaluate the predictive power of iNovo479 using comparisons of predicted and measured biomass yields for cultures grown on a set of individual organic substrates (Figure 1). By comparing experimental data with initial iNovo479 predictions, we found that the model predicts the relative amount of biomass produced with many individual carbon sources that are within 81–132% for mono-aromatic compounds and 212% for a model aromatic dimer of the values measured from laboratory cultures (Figure 1).

We also found that the type of lignin monomer (G-, S-, or H-) had an impact on biomass yield, as did the length and composition of any side chain on the aromatic ring. For example, vanillic acid and ferulic acid have the same ring structure but different side chains, resulting in varying biomass yields in iNovo479 simulations and experimental results. We propose that the observed increase in biomass yields reflects the use of aromatic side chains on substrates such as G-diketone or ferulic acid when compared to vanillic acid (see Section 3).

### 2.3. Testing Metabolism of the Dimer GGE

The observation that the experimental biomass yield when GGE is the sole carbon was about twice the experimentally observed yield prompted us to use iNovo479 to explore yield predictions depending on which monoaromatic derived from GGE is not used for microbial growth. When the β-*O*-4 ether bond in GGE is cleaved, guaiacol and hydroxypropiovanillone (HPV) are produced [11]. In iNovo479, the degradation of HPV proceeds via cleavage of the carbon side chain with production of vanillic acid, which enters the central metabolism for degradation of G-family aromatics [12], whereas guaiacol degradation follows *O*-demethylation to catechol and then aromatic ring cleavage of catechol with either an intradiol or an extradiol dioxygenase and production of non-aromatic intermediates that are further transformed to TCA intermediates. These pathways are consistent with predictions of aromatic metabolism in N. aromaticivorans DSM 12444 based on gene annotations in the KEGG database [13] and experimental observations in *Sphingomonas* [14,15] and *Sphingobium* [12]. The experimental yield measurements suggested that either HPV or guaiacol is not degraded in vivo. When guaiacol metabolism was removed from iNovo479, the predicted yield was 176 mgDW/mmol, and when HPV metabolism was removed, the predicted yield was 103 mgDW/mmol. Both yields were more consistent with the experimental observation of 133 mgDW/mmol (Figure 1) than our initially predicted value of 282 mgDW/mmol.

### 2.4. Testing the Impact of Demethylation Pathways on Biomass Production by iNovo479

The chemical difference between H-, G-, and S-family aromatics is in the number of methoxy groups on the aromatic ring (0, 1, and 2, respectively). In *N. aromaticivorans*, the first aromatic *O*-demethylation step is known to be catalyzed by one of the native tetrahydrofolate (THF)-dependent enzymes, DesA or LigM [6]. These enzymes transfer the methyl group from the aromatic ring to this cofactor [6]. In the case of S-type aromatics, the second *O*-demethylation step can be catalyzed by DesCD with the release of methanol and without any known cofactor [4] or LigM [6]. Given the differences in biomass yields that are observed or predicted by iNovo479 when cells are provided H-, G-, or S-family aromatics (e.g., *p*-hydroxybenzoic (*p*-HBA), vanillic, and syringic acids, respectively; Figure 1), we hypothesized that the enzyme used and resulting products of aromatic *O*-demethylation may contribute to biomass yield.

To test this hypothesis, we evaluated the ability of iNovo479 to predict biomass yields when reactions describing the native aromatic demethylases are replaced with other known enzymes that perform the demethylation without the THF cofactor and without the subsequent energy recovered in the form of NADH during folate co-factor recycling and oxidation of formate to carbon dioxide (Table 2 and Table S2). That is, the methoxy group is removed via the addition of oxygen instead of via a THF-dependent enzyme, and the one-carbon product of *O*-demethylation is converted to formate instead of proceeding through folate metabolism (Table S2). Under these simulations, iNovo479 predicts that the biomass yield from two methoxylated aromatics (vanillic and syringic acid) is reduced when the enzyme used for demethylation is no longer THF-dependent (9% and 16%, respectively), while there was no change in the predicted cellular yield from cells grown on *p*-hydroxybenzoic acid (0 methoxy groups) or glucose, which are not predicted to require *O*-demethylation (Table 2).

We next asked if the predictions of iNovo479 changed when the THF-dependent reactions for the native *N. aromaticivorans* aromatic demethylases were deleted and replaced with ones describing an enzyme that oxidizes NADH and produces formaldehyde as a consequence of aromatic demethylation, as in VanAB in *Pseudomonas putida* [16]. We were particularly interested in simulating this situation because of the recent discovery that *N. aromaticivorans* has an *O*-demethylase, DmtS, with a high degree of amino acid sequence identity to the VanA subunit of VanAB [6]. In simulations with a modified iNovo479 that includes VanAB-type demethylation, biomass yield predicted for growth on vanillic acid was reduced by 9% and yield from syringic acid was reduced by 16% in this scenario, the same amount seen in simulations with our hypothetical non-THF-dependent enzyme (Table 2).

### 2.5. Modeling Co-Metabolism of Biomass Aromatics Using iNovo479

**Figure 2:**
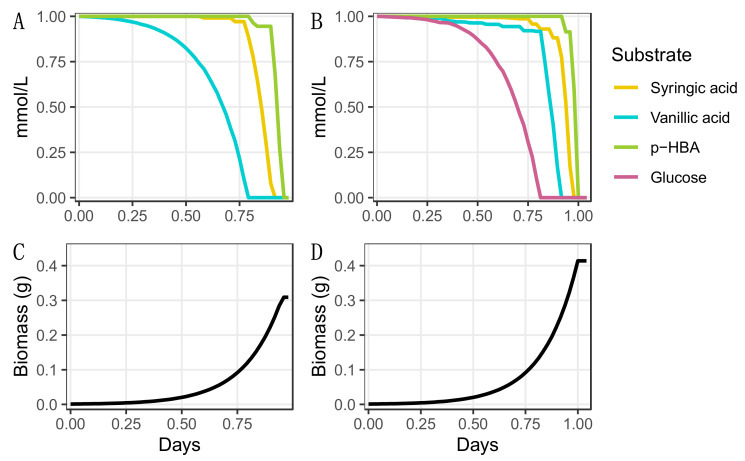
Co-metabolism of carbon sources predicted by iNovo479 for growth in the presence three individual aromatic substrates, syringic acid, vanillic acid, and *p*-hydroxybenzoic acid (*p*-HBA) without glucose (**A**,**C**) and with glucose (**B**,**D**). Each organic substrate was provided at 1 mmol/L.

The ability of *N. aromaticivorans* to funnel the mixed G-, H-, and S-monoaromatics that are present in deconstructed lignin into valuable products depends on the ability of this bacterium to co-metabolize multiple substrates. To evaluate the ability of iNovo479 to model co-metabolism of aromatic substrates, we provided the model with an equimolar mixture of vanillic acid, *p*-hydroxybenzoic acid, and syringic acid as sources of G-, H-, and S-monoaromatics and used dynamic flux balance analysis (dFBA) to simulate microbial growth. In this test, iNovo479 predicted that all three monoaromatics would be metabolized, and that vanillic acid would be consumed first, then syringic acid, and then *p*-hydroxybenzoic acid (Figure 2A,C). We also tested the ability of iNovo479 to model the co-metabolism of these same aromatics when an equimolar amount of a non-aromatic carbon source (glucose) is present. When glucose is also present in the simulation, the model predicts that glucose is consumed first, then vanillic acid, followed by syringic acid and finally *p*-hydroxybenzoic acid (Figure 2B,D).

We also ran the co-metabolism simulation on the versions of the model that used different O-demethylation enzymes (Table S2). In these simulations, *p*-HBA is predicted to be consumed before syringic acid (Figure S1). The change in the order of aromatic utilization in this simulation provides evidence of the energetic importance of the demethylation step in the observed pattern of substrate disappearance.

### 2.6. Testing if the Ratio of Aromatic to Non-Aromatic Carbon Source Impacts Biomass Production

**Figure 3:**
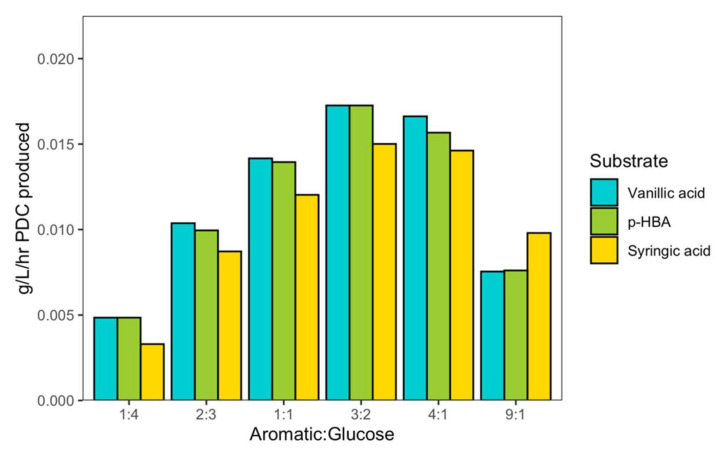
The ratio of aromatic:glucose for vanillic (VA), p-hydroxybenzoic acid (*p-*HBA), and syringic (SA) acid impacts on PDC production rate (g/L/h).

The growth of some engineered *N. aromaticivorans* strains requires the presence of another carbon source (e.g., glucose) if all the aromatic monomers in the deconstructed plant material are funneled towards the production of an aromatic-derived product such as PDC [4]. It is also likely that the deconstructed plant material used as a source of aromatics will contain other organic carbon substrates. Therefore, we wanted to predict how the ratio of aromatic to non-aromatic carbon sources would impact PDC production. To this end, we modified iNovo479 to allow extracellular PDC accumulation by removing enzyme reactions that simulate the gene deletions in the published PDC-producing strain [6] and performed dFBA with an aromatic substrate (vanillic acid, syringic acid, or *p*-HBA) and glucose in varying ratios. This analysis predicted that a three-to-two molar excess of aromatic to glucose was optimal for the highest rate of PDC production (Figure 3). We also noted that while syringic acid typically had the lowest rates of PDC production at all ratios, it out-performed vanillic acid and *p*-HBA when the aromatic substrate was in ninefold molar excess of glucose.

### 2.7. Using iNovo479 to Model Production of Additional Chemicals by Engineered N. aromaticivorans Strains

While the metabolic repertoire of *N. aromaticivorans* has yet to be analyzed in detail, its genome predicts it could convert aromatics into metabolites that could represent building blocks for a variety of potentially valuable chemicals. Thus, we also wanted to test the ability of iNovo479 to predict the synthesis of additional bioproducts that might be generated from aromatics by *N. aromaticivorans*. To this end, we performed dFBA using one or more “engineered” versions of the *N. aromaticivorans* GEM with added enzyme reactions needed to synthesize these products from intermediates that could be generated by metabolism of the three major aromatics found in deconstructed biomass (Table S3). The list of compounds tested in this study for production by *N. aromaticivorans* was taken from a technoeconomic analysis of a range of potential products (alcohols, organic acids, amines, and isoprenoids; Table 3) [17]. We focused on compounds that *N. aromaticivorans* could produce by the addition of three or fewer reactions to iNovo479.

In this set of dFBA simulations, we used 5 mmol/L vanillic acid as the only carbon source to evaluate whether iNovo479 would predict production of the chosen products directly from an aromatic substrate. These simulations were performed allowing for a range of substrate conversion to product (i.e., product yield), and for each product, we selected the yield that would sustain the highest product production rate (Table 3). This analysis predicted that all the evaluated products could be made by *N. aromaticivorans* from vanillic acid as the only carbon source. It also predicted products that could be produced only at small yields, with most of the substrate required to support microbial growth (e.g., ethanolamine with a yield of 0.06 mol-C_product_/mol-C_substrate_) and products for which a large fraction of the substrate could be directed to product formation (e.g., glutarate, cis-cis muconic acid (ccMA), citrate, and urea, all with yields greater than 0.4 mol-C_product_/mol-C_substrate_). In addition, the range of predicted production rates was large, from 0.0003 g/L/hr for ethanolamine production to 0.0048 g/L/h (16-fold higher) for urea.

Since the technoeconomic analysis of potential bioproducts [17] reported the value of the products (USD/kg) and the product titers (g/L) that could make microbial production feasible (i.e., a breakeven product titer; Table 3), we used this information and the optimal product yields predicted by iNovo479 (Table 3) to estimate the concentration of the vanillic acid aromatic substrate that would be necessary to yield individual chemicals at the breakeven product titers (Table 3). This analysis suggests that glutarate would require the lowest amount of vanillic acid to reach the breakeven product titer (54 g/L vanillic acid), whereas other products, such as acetate or ethanolamine, would require significantly higher vanillic acid concentrations to make microbial production feasible (1327 g/L and 5781 g/L vanillic acid, respectively).

More generally, the data derived from this set of simulations predicted that low-value products such as phenol and acetate would have a high substrate requirement even with moderate product yields (e.g., 0.49 mol-C_phenol_/mol-C_substrate_ and 0.80 mol-C_urea_/mol-C_substrate_, respectively). In contrast, chemicals such as ccMA, glutarate, citrate, acetaldehyde, and carotenoids such as zeaxanthin were predicted in these simulations to be advantageous products to generate from aromatics by engineered *N. aromaticivorans* strains due to the combination of moderate or high value and relatively high product yields (Table 3).

## 3. Discussion

The goal of this study was to use metabolic modeling to improve the ability to use *N. aromaticivorans* as a microbial chassis for producing valuable chemicals from the aromatics in deconstructed lignin. There are many considerations in the design and industrial use of microbes such as *N. aromaticivorans* for this purpose. On the input side, additional compounds might need to be added to depolymerized lignin to support microbial growth. For example, in addition to providing sources of nitrogen, sulfur, phosphorus, and trace metals, it might be required to provide non-aromatic organic carbon sources, if production of the desired product requires complete disruption of the aromatic catabolic pathways [4]. Several outputs of microbial lignin valorization must also be considered, including the need, value, and feedstocks required for engineered *N. aromaticivorans* strains to produce each compound. In addition, the thermodynamics of a native or engineered pathway must be balanced with the economics of feedstock deconstruction, the rate, yield, and value of the product, as well as the ease of product purification and the scalability of the overall process.

With all of these variables, identifying the optimal conditions for microbial lignin valorization can become a daunting task. Simulations from a GEM such as iNovo479 can reduce the number of conditions that need to be tested in the laboratory and can suggest experimental approaches that might not have been obvious based only on experimental data. A GEM can also be used to perform simulations on conditions that would be difficult to reproduce in the laboratory*,* such as extensive metabolic engineering or testing thermodynamically improbable reactions. This makes metabolic modeling a powerful approach to assist in building and improving the performance of a microbial lignin valorization pipeline. In this work, we developed a GEM of *N. aromaticivorans,* iNovo479, and used this GEM to simulate the metabolism and growth in the presence of numerous combinations of substrates, the impact of substrates in the deconstructed lignin feedstock, and the economic value of a set of potential bioproducts that could be produced by engineered *N. aromaticivorans* strains.

### 3.1. The Importance of Aromatic O-Demethylation

**Figure 4:**
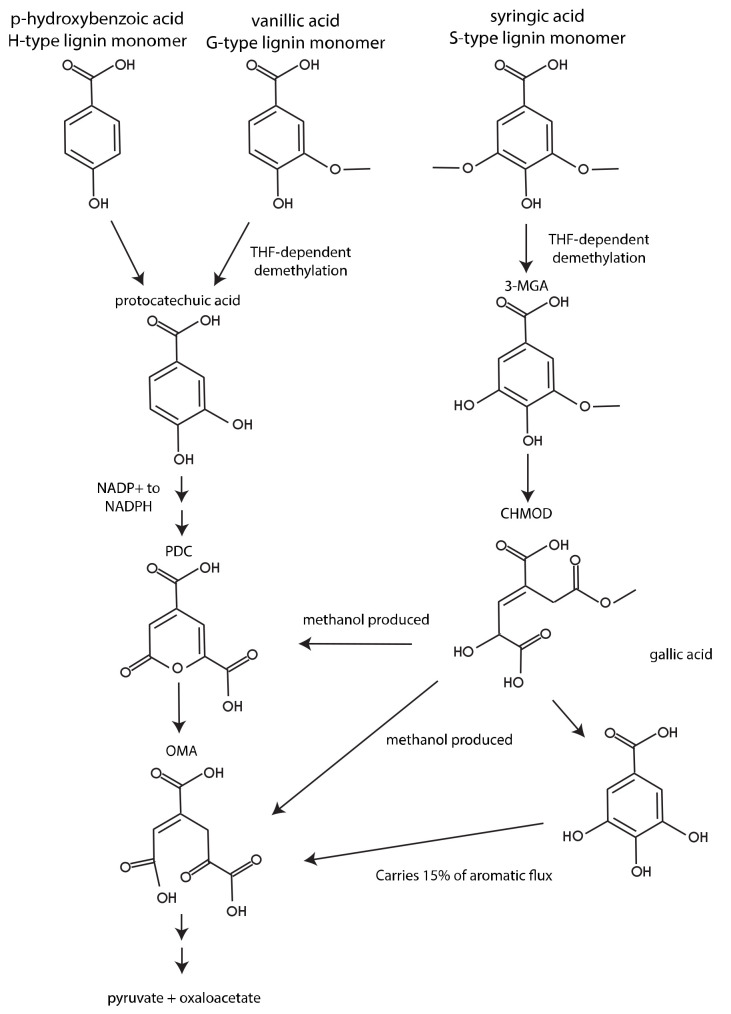
Aromatic metabolism of G-, S-, and H- aromatic compounds in *N. aromaticivorans*. Adapted from Perez et al. 2021 [6].

The three major lignin-derived monomers have 0, 1, or 2 methoxy groups on their aromatic rings, and it has been previously demonstrated that these methoxy groups have a large impact on how the aromatic ring is metabolized [4,7]. We used iNovo479 to illustrate the thermodynamic importance of how these methoxy groups are removed from aromatics. Predicted biomass yield from G-, S-, and H- aromatic monomers with identical side chains (vanillic acid, syringic acid, and *p*-HBA) varied, with syringic acid producing the most biomass and *p*-HBA producing the least (Figure 1). This is consistent with the amount of carbon available in each compound (Figure 4). However, we note that iNovo479 overestimated biomass yield from S-type monomers compared to experimental results. This may be due to an unknown aspect of aromatic metabolism that results in lower efficiency of conversion of S-type monomers to biomass. Alternatively, there may be an element of toxicity, either from the S-type compound themselves or from build-up of a byproduct of their catabolism, such as the predicted production of methanol as a byproduct of S-type aromatic metabolism (Figure 4). The ability of iNovo479 to accurately predict the metabolism and biomass yields of S-type aromatics would be increased by additional experiments to address the fate and impact of intermediates in the utilization of these compounds.

We then repeated the biomass yield simulations with alternative demethylation strategies, as the THF-dependent demethylation enzymes represent a significant energy source for microbial biomass production from aromatic substrates that contain 1 or 2 methyl groups (Table 2). Our simulations predicted that either replacing the native THF-dependent demethylation enzyme with a VanAB demethylase, such as that found in *P. putida,* or with a hypothetical, non-THF-dependent demethylase both resulted in a reduction in microbial biomass yield on aromatic compounds with 1 or 2 methoxy groups (vanillic and syringic acid) compared to either growth with *p*-HBA (no ring methoxy group) or when using the original THF-dependent demethylase. This effect was more pronounced with the VanAB demethylase than with the hypothetical demethylase for vanillic acid only, suggesting that the additional NADH cost associated with VanAB had a greater impact for vanillic acid than for syringic acid.

We next used dFBA to model co-metabolism of syringic acid, vanillic acid, and *p*-HBA. We found that vanillic acid was consumed first, then syringic acid, then *p*-HBA (Figure 2). While vanillic acid contains less carbon than syringic acid, an additional NADPH is produced during catabolism of vanillic acid and *p*-HBA, providing an advantage to a growing cell over syringic acid (Figure 4). Additionally, some of the carbon in syringic acid is lost as methanol. Syringic acid was still consumed before *p*-HBA, indicating that it is a more advantageous carbon source for biomass generation. However, these results changed when we repeated these dFBA simulations with the versions of iNovo479 with alternative demethylation pathways (Figure S1). With both the hypothetical non-THF-dependent demethylase and the VanAB demethylase, p-HBA was consumed before syringic acid. This suggests that the demethylation step itself is what conveys the increased biomass yield from syringic acid as compared to *p*-HBA.

### 3.2. Aromatic Side Chains Can Be a Help or a Hindrance to Microbial Biomass Yields

When provided each of the major aromatic monomer types, iNovo479 predicts differences in biomass yields (Table 2), some of which can be explained by the presence or absence of a side chain on the aromatic ring. iNovo479 predicts that aromatics containing a 3-carbon propene side chain (e.g., ferulic acid) or aromatic diketones (which contain a 2-carbon side chain) result in higher microbial biomass yields than those which lack a side chain (e.g., vanillic acid), and it predicts that this side chain is used as an additional source of carbon and energy after it is removed from the aromatic ring. This is the case with ferulic acid, which produces acetyl-CoA as part of its degradation and has 34% greater predicted microbial biomass yield and 32% greater actual biomass yield than vanillic acid, its counterpart with no side chain (Figure 1). However, G-diketone, which has the same chemical composition as ferulic acid, is predicted to produce 22% less microbial biomass than ferulic acid. We know that NADH oxidation by dehydrogenases is needed to reduce the side chains in aromatic diketones [18], so it is possible that the lower microbial biomass yield when using these compounds compared to one that contain a propene side chain is due to the higher thermodynamic energy requirement of metabolism of the diketone side chain. iNovo479 was only tested for performance when using aromatic compounds for which experimental evidence is available. However, the potential exists to use iNovo479 to predict the growth, biomass yields, and end products from a larger set of substrates once additional experimental evidence is available.

### 3.3. The Benefits of Co-Metabolism of a Non-Aromatic Carbon Source on Lignin Valorization

In addition to metabolizing a diverse set of native and chemically modified aromatics (e.g., aromatic diketones) that can be produced during oxidative lignin deconstruction, *N. aromaticivorans* is known [19,20] or predicted by its genome sequence [21] to use a diverse array of non-aromatic substrates, such as sugars or organic acids. Glucose has often been added to *N. aromaticivorans* cultures grown on aromatic monomers to boost microbial biomass production, yield of products such as PDC from aromatics, and growth rate [4]. In the case of the PDC-producing strains that convert nearly all aromatics to bioproducts, a equimolar ratio of aromatic to glucose has typically been used to support both cell growth and product accumulation [6]. However, supplementing a non-aromatic carbon source such as glucose would increase the cost of the lignin conversion process. Therefore, we sought to use iNovo479 to simulate yields of PDC with varying amounts of glucose and aromatic substrates to determine the optimal molar ratio. We found that a three-to-twofold molar excess of aromatic to a non-aromatic carbon source such as glucose provides sufficient microbial biomass for highest PDC production rate with each of the H-, G-, and S-family aromatics (Figure 3). Vanillic acid allowed the highest rate of PDC production, while syringic acid had the lowest rate. There was one exception to this—at high ratios of aromatic to glucose (nine-fold molar excess of aromatic), syringic acid outperformed both vanillic acid and *p*-HBA.

### 3.4. Using a GEM to Engineer Strains That Produce Additional Products from Lignin Aromatics

Another use of iNovo479 is to predict how genome engineering could be used to produce valuable products from lignin-derived aromatics. To test the feasibility of this approach, we added reactions to iNovo479 to simulate metabolism in *N. aromaticivorans* strains engineered to generate new products in this chassis microbe. Using this method, we found that iNovo479 predicted glutarate, zeaxanthin, ccMA, and citrate as candidates for production by *N. aromaticivorans* from plant aromatics given their predicted yields, the current market price per kilogram, and the high industrial demand for these compounds (Table 3). In addition, using further strain engineering, compounds such as ccMA and propanoate can be converted to other products to either increase their value (adipate and propionamide, respectively) [17,22] or to provide chemicals for additional markets.

The predictions of these modified versions of iNovo479 also provide insight into chemical features that can guide the selection of products to be generated from aromatics. For example, none of the high-value products predicted by these modified versions of iNovo479 contained nitrogen, possibly because each of the nitrogenous compounds we evaluated had relatively low prices per kilogram. In addition, in these simulations, the modified versions of iNovo479 became nitrogen-limited as the nitrogen demands of the bioproduct competed with the nitrogen demand of microbial biomass. Deconstructed lignin is not predicted to be rich in either ammonia or other nitrogenous compounds. The high energy cost of ammonia would also make the production of nitrogen-containing bioproducts from aromatics less likely at an industrial scale unless a low-cost and abundant source of nitrogen was identified.

The use of iNovo479 to predict compounds that could be derived from biomass aromatics by engineered *N. aromaticivorans* strains also illustrates the importance of choosing a product that is both connected to existing pathways and does not drain metabolites needed for other important metabolic pathways. For example, a product that requires many cofactors and metabolites for its synthesis, such as 1-hexadecanol, is predicted by modified versions of iNovo479 to be generated at low yield compared to metabolites derived from existing central carbon catabolic pathways (glycerol). However, using modified versions of iNovo479 to simulate the production of intermediates in microbial biomass synthesis, such as acetate, creates a competition for carbon between cell yield and product synthesis similar to that observed with some of the nitrogen-containing products we evaluated. Finally, iNovo479 predicts that synthesis of products such as 1-hexadecanol from aromatics via chain elongation is energy-intensive and likely to not be cost competitive since this pathway consumes large amounts of acetyl-CoA and NADPH.

While a GEM such as iNovo479 or its derivatives can model microbial metabolism, it is only one factor to consider when modeling lignin valorization at an industrial scale. For example, a complete technoeconomic analysis of lignin valorization must account for product need, price per kilogram, feedstock availability, cost of materials, equipment, and product purification when designing a microbial lignin valorization strategy for industrial use. Increasing production rates and product yields beyond the *in silico* predictions would likely rely on reactor operating conditions that allow higher biomass accumulation than observed in the simulations. This analysis illustrates the ability of a GEM such as iNovo479 to generate testable hypotheses for engineering a microbial chassis such as *N. aromaticivorans* to produce valuable chemicals from an abundant renewable resource such as deconstructed plant materials.

## 4. Materials and Methods

### 4.1. Building the GEM

iNovo479 was built manually using KEGG annotation data for *N. aromaticivorans* strain DSM 12444 plus reaction and compound IDs in the Integrated Microbial Genomes Database [13,23]. Gap filling of missing reactions was performed manually. The current instance of iNovo479 was assembled in XML format and run using COBRApy [24]. The model solver type was set to “glpk_exact” to eliminate small fluxes in inappropriate directions. Model quality was assessed using MEMOTE [25]. The overall MEMOTE score for iNovo479 was 85%, with a subtotal of 99% in the consistency category. The lower overall score is primarily due to the need to add custom reactions of aromatic metabolism that are not found in existing databases.

We also included a constraint in the model to simulate the known activity of side pathways in *N. aromaticivorans’* aromatic metabolism. This constraint is located in the catabolism of S-type aromatics, specifically the conversion of CHMOD to gallic acid. This pathway is modeled to operate at 15% of the flux through conversion of CHMOD to PDC in the wild-type version of the model based on experimental data [6]. This constraint is eliminated in the simulated PDC-producing strain, as this pathway was removed via gene deletions in the laboratory strain [6].

Transport reactions and the predicted energy costs (ATP hydrolysis and/or proton motive force contributions) associated with nutrient import were included in iNovo479, even where genes encoding those transporters have not been identified. For example, transporters for aromatic compounds have not been identified in *N. aromaticivorans*, but these compounds are hypothesized to enter the cell via TonB siderophore transporters in related sphingomonad bacteria [26,27]. The energy cost associated with aromatic transport encoded in iNovo479 is one molecule of ATP hydrolyzed and one H^+^ consumed by the proton motive force per substrate molecule imported, based on the described mechanism of TonB-dependent transport which uses proton motive force to cross the outer membrane and an ATP-dependent ABC transporter to cross the inner membrane [28]. While passive transport of lignin-derived compounds has been predicted in *Pseudomonas putida* [29], we chose to include energy costs for the transport of aromatic compounds as the more conservative approach for assembling iNovo479.

### 4.2. The Biomass Reaction

Genome annotation and laboratory studies predict that the α-proteobacterium *N. aromaticivorans* DSM 12444 is an obligate aerobe [30]. Consequently, we used the aerobic biomass reaction from iRsp1095 [10], a GEM of the α-proteobacterium *R. sphaeroides*, as the base of the iNovo479 biomass reaction and modified it to account for known differences in cell composition and metabolic activities between *R. sphaeroides* and *N. aromaticivorans* (Dataset S1).

The stoichiometry of the iNovo479 microbial biomass reaction was based on laboratory measurements of cellular protein, DNA, RNA, and phospholipid content. We measured the microbial biomass composition from duplicate 25 mL aerobic cultures grown on 2 mmol/L glucose and 2 mm/L vanillic acid as sole carbon sources in Standard Mineral Broth (SMB; DSMZ Medium 1185) [31] without tryptone or yeast extract. These cultures were grown in 100-mL flasks, shaken at 200 rpm and incubated at 30 °C. Cells from these cultures were centrifuged, freeze-dried, and weighed before other analyses were performed. Protein was measured after sonication of freeze-dried cells suspended in lysis buffer using the Qubit protein assay kit, while nucleic acids were extracted using phenol:chloroform and measured on a Qubit fluorometer (Thermo Fisher Scientific, Waltham, MA, USA). Phospholipids were extracted using chloroform and methanol followed by hot sulfuric acid, then quantified via spectrophotometric absorbance at 800 nm [10]. As no significant differences in biomass composition between cultures grown on glucose or vanillic acid were observed, the average of all cultures was used in assembling the biomass reaction for iNovo479.

Nucleotide amounts included in the biomass reaction were calculated based on the ratios of all four bases present in the genome [23] in combination with measurements of the cellular DNA and RNA. Amounts of amino acids included in the biomass reaction were calculated based on the predicted ratios of amino acids in annotated protein-coding regions of the genome [23] and the measured amount of cellular protein. Fatty acid composition was based on published data [30,32], using the cellular levels in aerobically grown cells from iRsp1095 [10]. Phospholipid composition data from *R. sphaeroides* were used in iNovo479 with appropriate modifications based on the lipid content and lack of lipopolysaccharide in *N. aromaticivorans*. Sphingoglycolipid-1, the most abundant sphingolipid in *N. aromaticivorans* [33], was added to iNovo479 to replace the lipopolysaccharides that are present in the iRsp1095 biomass reaction; its synthesis pathway was taken from the literature [34]. The resulting mass balance of the iNovo479 biomass reaction was 1.08 g per mmol of dry cell weight, close to the recommended value of 1.0 g of biomass per mmol dry cell weight [35] (Dataset S1). We then scaled values of metabolites in the biomass reaction so that its mass balance as included in the model was equal to 1.0 g of biomass.

Growth-associated maintenance included in the biomass reaction was derived from the measured amounts of DNA, RNA, and protein, plus ATP cost and non-growth-associated maintenance values under aerobic conditions used in iRsp1095 [10].

### 4.3. Microbial Biomass Yields

To measure microbial biomass yields in vivo and compare them to iNovo479 predictions, aerobic cultures were grown, in duplicate, in SMB with the indicated sole carbon sources provided at a concentration of 1 mmol/L. Cell density was measured using a Klett-Summerson photoelectric colorimeter with a red filter. We developed a standard curve to convert Klett units to grams of cell dry weight. One Klett unit (KU) is equivalent to 8 × 10^6^ CFU/mL for *N. aromaticivorans* [11]*,* while 100 KU is equivalent to 0.25 g of cell dry weight/L.

### 4.4. iNovo479 Modeling Conditions

Within the pathways modeled, iNovo479 includes reactions that require non-carbon nutrients such as ammonia, sulfur, iron, and phosphate. These compounds were provided as input fluxes in the amounts found in SMB. In addition, the iNovo479 GEM was encoded to provide unlimited influxes and outfluxes of oxygen, hydrogen ions, and water. Outfluxes of end product metabolites such as carbon dioxide and dead-end metabolites (compounds produced by the model that cannot be used for biomass and cannot be catabolized) were added to the model based on those found in *Escherichia coli* [36] and on the inability of the model to produce biomass without allowing outflux of a metabolite that could not be further catabolized.

To simulate growth under batch culturing conditions where substrate concentrations are not constant, we calculated maximum allowed uptake rates for organic and other substrates [16,37] using the following equation, where *K_s_* is the half-saturation constant (mmol/L), *V_max_* is the maximum velocity (mmol/min), *K_i_* is the inhibition constant (mmol/L), and *S* is the current substrate concentration (mmol/L):

(1) $$Uptake\;rate=V_{max}*\left(\frac{S}{\left(S+K_{s}\right)*\left(1+\frac{S}{K_{i}}\right)}\right)$$

*K_s_* and *V_max_* for glucose, vanillic acid, syringic acid, and *p*-hydroxybenzoic acid were approximated (Table S4) by fitting these parameters to growth curves of *N. aromaticivorans* grown in the presence of these organic substrates (Table 2). The parameters for these substrates were generalized to non-aromatic carbon substrates, G-type, S-type, and H-type aromatics. Maximum allowed uptake rates for ammonia, sulfate, and phosphate were taken from published values for other bacteria [38,39,40]. An inhibition constant (*K_i_*) was also included in iNovo479, as delayed growth is often observed when cells are initially grown in the presence of aromatics. Unless otherwise specified in the literature, the inhibition constant was assumed to be equal to the half-saturation constant.

The output of this equation was used as the maximum allowed uptake rate for substrate boundary reactions in the model, meaning that the model was permitted to choose an optimal flux between zero and the maximum allowed uptake rate for each. Typically, only one substrate boundary reaction was operating at its maximum allowed flux in any given iteration, indicating that uptake of this substrate was limiting biomass production.

We used dFBA [37], an iterative process where the output of one model run determines the input of the next cycle, to simulate growth of *N. aromaticivorans* under batch culture conditions. In this approach, for each cycle, steady-state simulations were performed with iNovo479, and after each run, steady-state fluxes of substrates and products and biomass production rates were recorded and used to modify the starting conditions for the next cycle. dFBA was run using a custom Python script and COBRApy [24].

### 4.5. Modeling Product Yields

Using the results of experiments with PDC-producing mutants of *N. aromaticivorans* [4,6], we created gene deletions in iNovo479 to simulate strains that accumulate PDC from aromatics. Because this modified PDC-producing version of the model would by default not use aromatic substrates if their route to central carbon metabolites is blocked, we first ran a wild-type version of iNovo479 model and recorded flux through the exchange reactions of the aromatic substrates. We then ran the PDC-producing version of the model with exchanges constrained to the flux observed in the wild-type iNovo479 model. This approach simulated the behavior of a strain that consumed aromatics and converted them to PDC, while using other organic substrates to support the synthesis of microbial cells, despite this scenario being non-optimal for microbial growth. In these simulations, we chose to use glucose as the non-aromatic carbon source since this sugar is known to be rapidly metabolized by *N. aromaticivorans* and has been used to support the production of PDC from aromatics by engineered strains [4].

Modeling the accumulation of product compounds not already included in the initial iNovo479 version required the addition of enzymatic reactions to the GEM (Table S3). Additional reactions from KEGG were added to allow production of the desired compound and bioproduct accumulation was tied to a fixed percentage of substrate uptake, reducing competition with biomass. This simulates the activity of an overexpression-type strain, where the enzymes for a desired pathway are over-produced to allow increased flux through that pathway.

The GEM iNovo479 and its derivatives are publicly available for use at github.com/GLBRC/iNovo/ (accessed on 13 April 2022). All code used in this research is also available at this link, along with instructions for its use. We hope that iNovo479 will be a resource for the research community.

## 5. Conclusions

In this research, we built a GEM for *N. aromaticivorans* and demonstrated its potential to make testable predictions about aromatic metabolism and the genetic modifications needed to improve microbial lignin valorization. We demonstrated the thermodynamic favorability of demethylating aromatic methoxy groups by specific enzyme types and of capturing the carbon and energy of the organic side chains that naturally exist on ferulic acid and other lignin aromatics. By modeling production of one valuable compound, PDC, iNovo479 predicts that reduced levels of non-aromatic carbon will allow increased rates of synthesis of this chemical. Finally, we combined iNovo479 predictions with a published technoeconomic analysis to suggest a set of priority candidates for microbial lignin valorization by *N. aromaticivorans* and make predictions on product characteristics that are likely to enhance acceptance by the industry. The current instance of iNovo479 includes reactions for the metabolism of aromatic compounds for which there is some enzymatic or genetic evidence in this or related bacteria. We have not attempted to use it to predict the utilization of aromatic compounds which have either not been tested as growth substrates or for which experimental evidence is lacking for their metabolism. We predict that the lessons learned from assembly and use of iNovo479 will aid useful future studies of biological lignin valorization and efforts to generate other valuable chemicals from *N. aromaticivorans* and other engineered microbes.

## Figures and Tables

**Table 1:** Contents of iNovo479.

Characteristic	Value
Number of reactions	645
Number of metabolites	604
Number of genes	479
Number of reactions without genes	135
Number of cell compartments	3 ^1^
Number of transformation reactions	549
Number of transport reactions	61
Number of exchange reactions	21
Number of demand reactions	12

^1^ iNovo479 includes reactions predicted to take place in the cytoplasmic, periplasmic, and extracellular compartments.

**Table 2:** Biomass yield (mg dry weight/mmol carbon source) predictions in the presence and absence of the native *N. aromaticivorans O-*demethylases or when this reaction is replaced by a VanAB *O*-demethylase reaction.

Carbon Source	With Native Aromatic *O*-Demethylases	Hypothetical Non-THF-Dependent *O*-Demethylases	With Native *O*-Demethylases Replaced with VanAB *O*-Demethylase
Glucose	114	114	114
Vanillic acid	114	104	104
Syringic acid	116	97	97
*p*-Hydroxybenzoic acid	88	88	88

**Table 3:** iNovo479 prediction of product yields that would maximize product production rates from vanillic acid as the only carbon source, and vanillic acid concentrations needed to obtain breakeven product titers.

Product	Product Value (USD/kg) ^1^	Product Breakeven Titer (g/L) ^1^	Predicted Product Yield that Sustains Maximum Production Rates (mol-Cproduct/mol-Csubstrate) ^2^	Predicted Maximum Production Rate (g/L/h) ^3^	Vanillic Acid Concentration Needed to Reach Product Breakeven Titer (g/L) ^4^
Glutarate	10.76	17	0.40	0.0021	54
Zeaxanthin	10.00	18	0.05	0.0009	106
cis-cis muconic acid	1.81	94	0.57	0.0034	195
Acetaldehyde	2.21	85	1.27	0.0019	255
Glycerol	1.37	145	1.0	0.0032	265
Citrate	1.21	166	0.42	0.0041	338
1-Hexadecanol	2.01	69	0.14	0.0013	342
Butanoate	1.50	130	0.64	0.0020	388
Urea	0.31	832	3.94	0.0048	491
Phenol	0.86	145	0.49	0.0015	529
Propanoate	2.09	89	0.33	0.0016	612
Acetate	0.60	379	0.80	0.0024	1327
Ethanolamine	1.54	126	0.06	0.0003	5781

^1^ Data adapted from Wu et al., 2018 [17]. Products included in this table are limited to those that required three or fewer additional reactions to simulate their production by iNovo479. ^2^ From iNovo479; product yield (moles of carbon in product per moles of carbon in substrate) set up in the product flux exchange that maximized predicted production rate. ^3^ From iNovo479, resulting from selected product yield. ^4^ Vanillic acid was used in the simulations as a proxy for the amount of aromatic that would need to be obtained from deconstructed plant biomass to reach the desired product breakeven titer.

## Data Availability

All data, code, and model builds used in this paper are available at github.com/GLBRC/iNovo/ (accessed on 13 April 2022). Input KEGG reaction data for these models are available in the Integrated Microbial Genomes database with taxon ID 640427126.

## Supplementary Materials

The following supporting information can be downloaded at: https://www.mdpi.com/article/10.3390/metabo12040366/s1, Figure S1. Co-metabolism of SA, VA, and p-HBA, Table S1. Measured biomass composition of *N. aromaticivorans*; Table S2. Native vs. hypothetical vs. *P. putida* demethylation reactions in iNovo479; Table S3. Reactions added to iNovo479 to simulate engineered strains capable of producing non-native chemicals; Table S4. Kinetic constants used to simulate maximum allowed substrate uptake rates, Dataset S1. Biomass equation calculations.
