# Supplementary material for: iNovo479: Metabolic Modeling Provides a Roadmap to Optimize Bioproduct Yield from Deconstructed Lignin Aromatics by Novosphingobium aromaticivorans
Source: Metabolites. 2022 Apr 18;12(4):366. doi: 10.3390/metabo12040366 (PMC9028409; doi:10.3390/metabo12040366)
Supplement: Supplementary file 1 [file metabolites-12-00366-s001.zip › Supplemental_Files/iNovo_supplemental_2022-03-18.pdf]

iNovo479: Metabolic Modeling Provides a Roadmap to Optimize Bioproduct Yield from deconstructed Lignin Aromatics by *Novosphingobium aromaticivorans*

Alexandra M. Linz, Yanjun Ma, Samuel Scholz, Daniel R. Noguera and Timothy J. Donohue

Supplemental information

Table S1. Measured biomass composition of *N. aromaticivorans*

Characteristic	Value[1]
**DNA**	1.7%
**RNA**	6.6%
**Protein**	60.0%
**Phospholipids**	7.3%

[1] Percent of total biomass

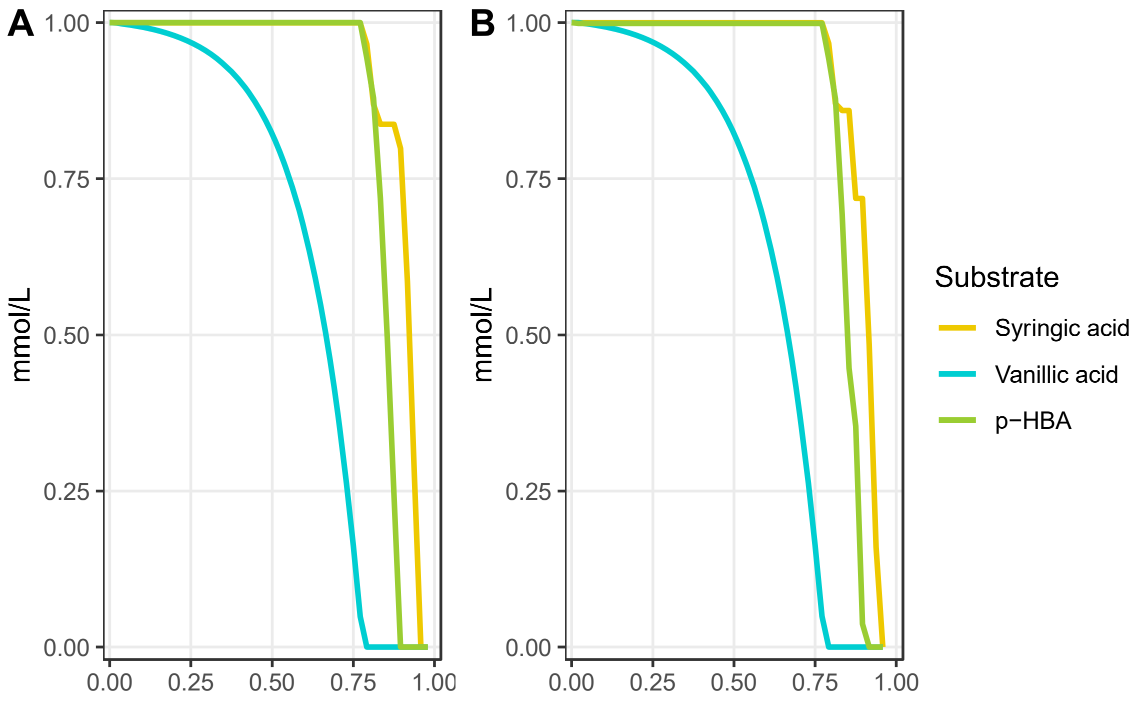

Figure S1. Co-metabolism of SA, VA, and p-HBA by versions of iNovo479 with a hypothetical, non-THF dependent demethylation step (Panel A) and with a VanAB-like demethylation step (Panel B). Each organic substrate was provided at 1 mmol/L.

Table S2. Native vs. hypothetical vs. *P. putida* demethylation reactions in iNovo479

Model version	Reaction ID	Reversible	Reaction Name	Reaction
**native**	A007	NO	Vanillic acid demethylation 1	Vanillic acid + THF => 5-Methyltetrahydrofolate + PCA
**native**	A008	NO	Vanillic acid demethylation 2	5-Methyltetrahydrofolate + NAD+ => NADH + 5,10-Methylenetetrahydrofolate + H+
**native**	R01220	YES	5,10-methylenetetrahydrofolate:NADP+ oxidoreductase	5,10-Methylenetetrahydrofolate + NADP+ <=> 5,10-Methenyltetrahydrofolate + NADPH
**native**	R01655	YES	5,10-Methenyltetrahydrofolate 5-hydrolase (decyclizing)	5,10-Methenyltetrahydrofolate + H2O <=> 10-Formyltetrahydrofolate + H+
**native**	R00944	NO	10-Formyltetrahydrofolate amidohydrolase	10-Formyltetrahydrofolate + H2O => formate + THF
**native**	A018	NO	Syringic acid demethylation	Syringic acid + THF => 5-Methyltetrahydrofolate + 3-methylgallate
**hypothetical**	altA007 (replaces A007)	NO	Hypothetical vanillic acid demethylation 1	Vanillic acid + O2 => formate + PCA
**hypothetical**	altA018 (replaces A018)	NO	Hypothetical syringic acid demethylation	Syringic acid + O2 => formate + 3-methylgallate
P. putida	ppA007 (replaces A007)	NO	*P. putida* vanillic acid demethylation	Vanillic acid + NADH + O2 + H+ => NAD+ + H2O + formaldehyde + PCA
P. putida	ppA018 (replaces A018)	NO	*P. putida* syringic acid demethylation	Syringic acid + NADH + O2 + H+ => NAD+ + H2O + formaldehyde + 3-methylgallate

Table S3. Reactions added to iNovo479 to simulate engineered strains capable of producing non-native chemicals.

**Reaction ID**	**Reversibility**	**Name**	**Bioproduct**	**Reaction**
**engR01704**	Reversible	Palmitaldehyde:NAD+ oxidoreductase	1-hexadecanol	Hexadecanal + NAD+ + H2O <=> Hexadecanoic acid + NADH + H+
**engR02462**	Reversible	Hexadecanol:NAD+ oxidoreductase	1-hexadecanol	1-Hexadecanol + NAD+ <=> Hexadecanal + NADH + H+
**engR01976**	Reversible	(S)-3-Hydroxybutanoyl-CoA:NADP+ oxidoreductase	Butanoic acid	(S)-3-Hydroxybutanoyl-CoA + NADP+ <=> Acetoacetyl-CoA + NADPH + H+
**engR03026**	Reversible	(S)-3-hydroxybutanoyl-CoA hydro-lyase	Butanoic acid	(S)-3-Hydroxybutanoyl-CoA <=> Crotonoyl-CoA + H2O
**engR01171**	Reversible	butanoyl-CoA:NAD+ trans-2-oxidoreductase	Butanoic acid	Butanoyl-CoA + NAD+ <=> Crotonoyl-CoA + NADH + H+
**engR01179**	Reversible	butanoyl-CoA:acetate CoA-transferase	Butanoic acid	Butanoyl-CoA + Acetate <=> Butanoic acid + Acetyl-CoA
**engR00449**	Irreversible	L-lysine:oxygen 2-oxidoreductase (decarboxylating)	Glutarate	L-Lysine + Oxygen => 5-Aminopentanamide + CO2 + H2O
**engR02273**	Irreversible	5-aminopentanamide amidohydrolase	Glutarate	5-Aminopentanamide + H2O => 5-Aminopentanoate + Ammonia
**engR02274**	Reversible	5-aminopentanoate:2-oxoglutarate aminotransferase	Glutarate	5-Aminopentanoate + 2-Oxoglutarate <=> 5-Oxopentanoate + L-Glutamate
**engR02401**	Reversible	glutarate-semialdehyde:NAD+ oxidoreductase	Glutarate	5-Oxopentanoate + NAD+ + H2O <=> Glutarate + NADH + H+
**engR00841**	Irreversible	sn-glycerol-3-phosphate phosphohydrolase	Glycerol	sn-Glycerol 3-phosphate + H2O => Glycerol + Orthophosphate
**engR00728**	Reversible	L-tyrosine phenol-lyase (deaminating; pyruvate-forming)	Phenol	L-Tyrosine + H2O <=> Phenol + Pyruvate + Ammonia

Table S4. Kinetic constants used to simulate maximum allowed substrate uptake rates

	Half-saturation constant (K_s)(mmol/L)	Substrate velocity (V_{max})(mmol/min)	Inhibition constant (K_i)(mmol/L)
Non-aromatic carbon substrates	0.139	0.500	0.139
S-type aromatics	0.050	0.582	0.050
H-type aromatics	0.050	0.902	0.050
G-type aromatics	0.100	0.569	0.100
Ammonia	0.100	0.500	0.100
Phosphate	0.002	0.060	0.100
Sulfate	0.003	0.002	0.100
Iron	0.003	0.002	0.100
